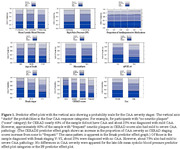# Late‐life Blood Pressure and Cerebral Amyloid Angiopathy among Community‐based Older Adults: An Analysis using the U.S. National Alzheimer’s Coordinating Center Uniform Data Set

**DOI:** 10.1002/alz.088475

**Published:** 2025-01-09

**Authors:** Mo‐Kyung Sin

**Affiliations:** ^1^ Seattle University, Seattle, WA USA

## Abstract

**Background:**

Cerebral amyloid angiopathy (CAA) and hypertension are the two most common risk factors of intracranial hemorrhage leading to cognitive impairment, but less is known about how the two relate. A better understanding of the association between these risk factors is a key step towards developing new strategies to manage hypertension and attenuate CAA progression.

**Method:**

This study analyzed data from 2,510 participants in the National Alzheimer’s Coordinating Center (NACC) dataset who had CAA and longitudinal blood pressure (BP) measurements before death. The presence of CAA (autopsy‐based) was determined on immunohistochemistry and categorized as none, mild, moderate, or severe. Multivariable proportional odds logistic regression was used to examine the associations between CAA stages and BP. Systolic BP (SBP) and pulse pressure (PP) were modeled as primary predictors. Covariates also included age at death, sex, antihypertensives, microinfarcts, APOE e4, CERAD, and Braak stages.

**Result:**

CAA was present in 1,580 (62.9%) participants; 759 (30.2%) mild, 529 (21.1%) moderate, and 292 (11.6%) severe. Neither late‐life mean SBP ≥140mmHg nor (PP) ≥ 50mmHg were associated with CAA stages. Participants with APOE e4 allele (adjusted odds ratio (aOR) = 1.80, 95% CI: 1.53‐2.11) and males (aOR = 1.25, 95% CI: 1.08, 1.46) had significantly higher odds of having more advanced CAA stage than those who were APOE e4 noncarriers and females. The odds of more severe CAA increased with higher Braak and CERAD stages: Braak stage III‐IV (aOR = 1.51, 95% CI: 1.17‐1.95), Braak stage V‐VI (aOR = 2.43, 95% CI: 1.82, 3.23), CERAD mild (aOR = 2.73, 95% CI: 2.0, 3.60), CERAD moderate (aOR = 3.46, 95% CI: 2.61,4.60), and CERAD frequent (aOR = 5.25, 95% CI: 3.90, 7.09). Figure 1 shows the effect plots for all the variables included in the model.

**Conclusion:**

High SBP (≥140 mmHg) and PP (≥50 mmHg) had no effect on CAA severity stage. Considering CAA and hypertension being major risk factors for intracranial hemorrhage, hypertension may have a synergistic effect on intracranial hemorrhage development in people with CAA positive. More empirical studies are needed to confirm the finding.